# Quantifying the Potential Bias when Directly Comparing Standardised Mortality Ratios for In-Unit Neonatal Mortality

**DOI:** 10.1371/journal.pone.0061237

**Published:** 2013-04-05

**Authors:** T. Alun Evans, Sarah E. Seaton, Bradley N. Manktelow

**Affiliations:** Department of Health Sciences, University of Leicester, Leicester, United Kingdom; Tehran University of Medical Sciences, Iran (Islamic Republic of Iran)

## Abstract

**Introduction:**

The Standardised Mortality Ratio (SMR) is increasingly used to compare the performance of different healthcare providers. However, it has long been known that differences in the populations of the providers can cause biased results when directly comparing two SMRs. This is potentially a particular problem in neonatal medicine where units provide different levels of care.

**Methods:**

Using data from The Neonatal Survey (TNS), babies born at 24 to 31 weeks gestational age from 2002 to 2011 and admitted to one of 11 UK neonatal units were identified. Risk-adjusted SMRs were calculated for each unit using a previously published model to estimate the expected number of deaths. The model parameters were then re-estimated based on data from each individual neonatal unit (“reference” unit) and these then applied to each of the other units to estimate the number of deaths each unit would have observed if they had the same underlying mortality rates as each of the “reference” hospitals. The ratios of the SMRs were then calculated under the assumption of identical risk-specific probabilities of death.

**Results:**

7243 babies were included in all analyses. When comparing between Network Neonatal Units (Level 3) the ratio of SMRs ranged from 0.92 to 1.00 and for the comparisons within Local Neonatal Units (Level 2) ranged from 0.79 to 1.56. However when comparing between neonatal units providing different levels of care ratios up to 1.68 were observed.

**Conclusions:**

If the populations of healthcare providers differ considerably then it is likely that bias will be an issue when directly comparing SMRs. In neonatal care, the comparison of Network Neonatal Units is likely to be useful but caution is required when comparing Local Neonatal Units or between units of different types. Tools to quantify the likely bias are required.

## Introduction

For at least 150 years [1] there have been attempts to collect, analyse and compare data on clinical outcomes between units. In the last few decades the comparison of outcomes between different healthcare providers (e.g. units, surgeons, GPs) has increasingly been systematically implemented [2]. There are many reasons for performing direct comparisons between healthcare providers: (i) to help with the identification of poor performing providers so that it is possible for them to be investigated and for improvements to be made if necessary; (ii) to identify centres of excellence and share best practice; (iii) if policy makers believe that competition will improve performance they need patients and general practitioners to choose between units and this can only be done if information that allows comparison is available [3]; (iv) if the trend towards payment by performance [4], [5] is to be fair it is important to have a system of comparison that is reliable and trustworthy.

The need to adequately adjust outcomes for differences in case mix (risk-adjustment) is well documented [6]. A unit or clinician tending to treat only those patients with good prognoses would be expected to have a high rate of ‘good’ outcomes whilst, conversely, those treating patients with poor prognoses would expect a high rate of ‘poor’ outcomes. The Standardised Mortality Ratio (SMR) is the most widely used summary statistic for binary outcomes (e.g. death, post-operative infection) to report case-mix adjusted outcomes for healthcare providers [7]. The SMR is an indirectly standardised measure of outcome and is defined as the ratio of the observed number of events to the number expected given the case-mix profile of the patients. The expected number of events is calculated using the observed probability of the event in a larger reference population (e.g. regional or national data). The SMR is often used as a hospital-wide measure, such as the Hospital Standardised Mortality Ratio (HSMR) [8], [9] and the Summary Hospital Mortality Indicator (SHMI) [10]. Additionally, and perhaps more informatively [11]–[13], SMRs are also often reported by clinical sub-specialties, for example, cardiac surgery [14] and neonatal survival [15].

The SMR is, therefore, a measure of how the outcomes for an individual healthcare provider compared with those of a reference population: e.g. how Unit A's patients did compared with how they would be expected to have done if Unit A had performed at national rates. It has long been recognised that directly comparing the SMRs of two different healthcare providers may be inappropriate as the SMRs for two providers performing equally for each patient type will not necessarily take the same value if the providers' population structures are different [16]–[18]: “[the SMR] *is only a* ‘*single-pair*’ *method*, *and if it is applied to a number of groups it may only be thanks to the mercy of Providence that it is not grossly misleading*” [19]. On the other hand, there are others who argue that any bias that arises when comparing two SMRs is likely to be small and would not adversely affect any inferences drawn [20], [21]. Although it is unclear how much bias would really occur in practice, measures such as the HSMR and SHMI are increasingly being used to compare hospitals that potentially have patient populations with very different sets of risk factors.

This bias might raise particular problems in the direct comparison of SMRs where there are large differences in the types of patient treated in the healthcare providers. This is seen in neonatal care where in the United Kingdom (UK) neonatal units are organised into networks with different units providing different levels of care [22], [23]. This organisation of care is similar to the pattern of neonatal care seen in most other countries [24]–[26]. Within each network there are *Special Care Units* (Level 1) which provide special care but do not aim to provide any continuing high dependency or intensive care, *Local Neonatal Units* (Level 2) which provide high dependency care and some short-term intensive care as agreed within the network and at least one *Network Neonatal Unit* (Level 3) which provides the whole range of medical neonatal care (but not necessarily all specialist services such as neonatal surgery) [22]. The case-mix for individual units, therefore, reflects local policies on care, admission and transfer and is likely to vary both within and between networks, meaning that it may be inappropriate to directly compare SMRs between neonatal units, particularly units providing different levels of care.

In this paper we will describe the source of this potential bias and will quantify the bias which may arise when comparing SMRs for in-unit deaths of very preterm babies (born from 24 to 31 weeks gestational age) admitted for neonatal care in the UK. We were unable to find any previous examples of the potential size of this bias in any clinical specialty. A suggested method for quantifying the differences in case-mix between two populations, the M-statistic, will be examined to investigate the size of the bias and the value of this statistic.

## Methods

### Ethics Statement

The Neonatal Survey has been given permission to collect data by the Patient Information Advisory Group (now the National Information Governance Board for Health and Social Care).

### Standardised mortality ratio

The SMR is the indirectly standardised ratio of the observed number of events *O* to the number expected *E* calculated using the proportion of events occurring in a reference population: i.e.
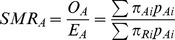



where *π_Ai_* is the probability that the event will occur for an observation in the *i*th case-mix stratum in Provider A; *π_Ri_* is the probability that the event will occur for an observation in the *i*th case-mix stratum in the reference population; *p_Ai_* is the proportion of observations in the *i*th case-mix stratum in Provider A.

Therefore, for the SMRs for two providers (Provider A & Provider B) to be equal the following would need to be true:
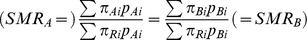



However, even if the stratum-specific event probabilities were identical for both providers for all strata (i.e. *π_Ai_* = *π_Bi_* = *π_ABi_* for all values of *i*), and different from the reference population for at least one stratum, the SMRs would only be sure to take the same value if their population structures were also the same (i.e. *p_Ai_* = *p_Bi_* for all values of *i*):
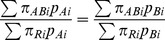



In other words, even if two healthcare providers were performing identically for each type of patient, their SMRs would not be the same value if the proportion of each patient type was different for each provider (i.e. *p_A_* ≠*p_Bi_* for at least one value of *i*). The size of any difference between the SMRs of two providers with the same stratum-specific event probabilities depends on the size of the difference between the risk-specific mortality in the units (*π_ABi_*) and that of the reference population (*π_Ri_*) and on the size of difference in population structure between the units (*p_Ai_* and *p_Bi_*).

### Data

Data were obtained from The Neonatal Survey (TNS), a population-based audit of in-patient neonatal care based in the East Midlands and Yorkshire Regions of the UK [27]. All neonatal services in the regions contribute to TNS and neonatal units in adjacent regions also permit data collection on eligible babies. The present survey was established in 1990 and now covers an area which has around 120,000 births each year with information collected on all babies admitted to neonatal care who are born at less than 33 weeks gestational age to mothers resident in the study area. Seven part-time neonatal nurses prospectively collect the data during regular visits to the neonatal units, with audits and validation checks undertaken to ensure data collection is complete. Information is collected on antenatal, perinatal and neonatal factors including gestational age, gender, and birthweight. For this paper, data were included for the 11 neonatal units that contributed to TNS from 2002 to 2011: three Network Neonatal Units and eight Local Neonatal Units. The two Special Care Units which contributed data to TNS were not included as they were not expected to provide care to babies at risk of death.

Data were extracted for all preterm babies admitted to neonatal care who were born from 2002–2011 at 24 to 31 completed weeks gestational age. Observations were excluded for missing or implausible birthweight; missing or ambiguous gender and inevitably lethal congenital anomalies.

Approximately 28% of very preterm babies recorded in TNS are transferred between neonatal units during their period of neonatal care. In this paper the ‘unit of care’ was defined as the unit that the baby was a patient in at the end of their third day of life or the unit of death if the baby died before the end of the third day of life. It is expected that by the third day of life a baby's level of risk will have been fully assessed by the clinician team, the baby will have been stabilised and transferred to the unit which can provide the most appropriate care.

### Estimating bias

An SMR was calculated for each neonatal unit by dividing the observed number of deaths by the expected number of deaths estimated using a published mortality model and parameters estimates derived using TNS data [28]:
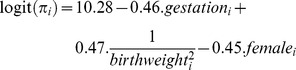



A 95% confidence interval was calculated using the normal method [29].

In order to estimate the likely bias that would arise solely from the differences in case-mix when directly comparing SMRs from two different neonatal units, pair-wise comparisons were made assuming that the observed risk-specific mortality was identical for the two units. This was done by using the data of each unit in turn to calculate new ‘reference’ unit specific estimates for the parameters of the model, i.e. for Unit A:
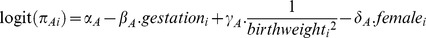



These estimates were then applied to each of the other units to estimate the number of deaths that would have been observed if their risk-specific mortality was the same as the ‘reference’ unit and an SMR was then calculated for each of the other units. Any difference between these SMRs must be due to the differences in the units' population structures and the ratio of the SMRs was calculated to quantify the size of this difference. Therefore applying the observed mortality probabilities from unit A to unit B gives the ratio
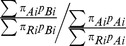



Since the observed number of events for each unit was estimated using the same case-specific mortality for each of the pair-wise comparisons, the SMRs for the two neonatal units would be expected to be equal if there were no differences in the case-mix structure.

### M-statistic

The M-statistic is a measure of agreement between two populations and was calculated in this analysis using the methods, and ranges, outlined by Boyd *et al* [30]. The proportion of patients in each unit with a predicted survival in each of following six ranges was calculated: *π_i_*≥0.96; 0.91≤*π_i_*<0.96; 0.76≤*π_i_*<0.91; 0.51≤*π_i_*<0.76; 0.26≤*π_i_*<0.51; *π_i_*<0.26.

The M-statistic of any particular pair of units is the sum of the lower of the two units' proportions in each range:
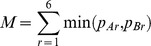



where *p_Ar_* and *p_Br_* are the proportion of babies in range *r* for units A and B respectively.

An M-statistic of 1 is achieved if the proportions are identical for every range, the lower the M-statistic the more divergent the populations. The M-statistic was calculated for each pair-wise comparison and then plotted against the ratio of SMRs to examine the relationship between each pair of units.

## Results

In total 7,340 eligible babies were identified from TNS. Babies were excluded for lethal congenital anomalies (n = 78, 1.1%); missing or implausible birthweight (n = 6, 0.1%); missing or indeterminate gender (n = 13, 0.2%). Therefore, 7,243 babies were included in the analyses.

The Neonatal Network Units admitted more babies than the Local Neonatal Units, and these babies tended to be born at earlier gestational ages, have lower mean birthweight and were more likely to die before discharge. The Local Neonatal Units varied widely in the number of babies admitted and the proportion of deaths (Table 1). The estimated SMRs for the units ranged from 0.54 to 1.28 with variation for both types of units. The confidence intervals for three units (C, F and I) did not include the value 1 (Table 2).

**Table 1:** Descriptive statistics of babies by unit of care.

	Number of Babies	Died before discharge Number (%)	Gestation Mean (s.d.)	Birthweight Mean (s.d.)	Gender (female %)
***Network Neonatal Units***					
**A**	1233	115 (9.3%)	29.0 (2.2)	1205 (368)	44.9%
**B**	1676	210 (12.5%)	28.7 (2.2)	1161 (377)	47.1%
**C**	1464	239 (16.3%)	28.4 (2.2)	1104 (364)	48.3%
***Local Neonatal Units***					
**D**	236	11 (4.7%)	29.8 (1.6)	1347 (331)	43.2%
**E**	313	18 (5.8%)	29.5 (1.7)	1291 (317)	47.9%
**F**	474	14 (3.0%)	29.7 (1.7)	1343 (342)	46.4%
**G**	315	20 (6.3%)	29.4 (1.9)	1282 (351)	48.2%
**H**	321	23 (7.2%)	29.7 (1.8)	1310 (331)	48.3%
**I**	648	82 (12.7%)	29.1 (2.1)	1230 (384)	44.3%
**J**	323	32 (9.9%)	29.3 (2.0)	1245 (376)	48.0%
**K**	240	20 (8.3%)	29.4 (1.9)	1287 (357)	45.8%

**Table 2:** SMRs of units with 95% normal confidence intervals.

	SMR	95% confidence interval
***Network Neonatal Units***		
**A**	0.92	(0.77 to 1.07)
**B**	1.06	(0.95 to 1.18)
**C**	1.21	(1.09 to 1.32)
***Local Neonatal Units***		
**D**	0.91	(0.38 to 1.44)
**E**	0.91	(0.51 to 1.31)
**F**	0.54	(0.18 to 0.89)
**G**	0.85	(0.49 to 1.22)
**H**	1.18	(0.78 to 1.58)
**I**	1.28	(1.07 to 1.49)
**J**	1.17	(0.84 to 1.50)
**K**	1.17	(0.74 to 1.60)

**Figure 1:**
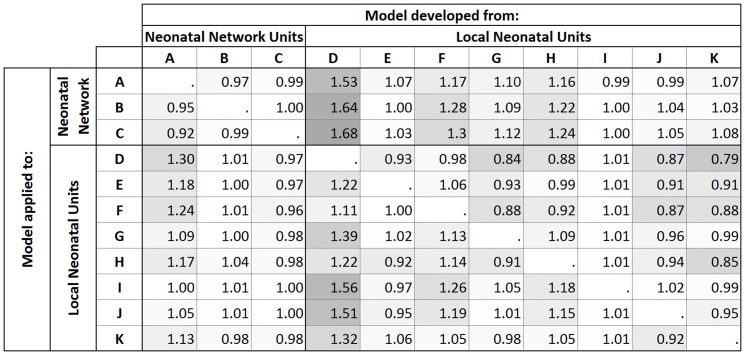
The ratio of SMRs assuming the same observed risk-specific probability of death in each pair of neonatal units.

The values of the ratios of SMRs obtained when assuming the risk-specific mortality probabilities are equal in pairs of units are given in Figure 1: the darker shading represents values of the ratio furthest from 1. Overall, the values for the ratio of the SMRs ranged from 0.79 to 1.68. When applying the risk-specific mortality of one Neonatal Network Unit to another Neonatal Network Unit the ratios of the SMRs ranged from 0.92 to 1.00 and for the comparisons within Local Neonatal Units the ratios ranged from 0.79 to 1.56.

The values for ratios obtained by applying the risk-specific probabilities from Unit I to other units were particularly close to 1 (0.99 to 1.01). The estimates for the model parameters obtained using data from Unit I alone were similar to values in the full model: *α_I_* = 9.92; *β_I_* = −0.44; *γ_I_* = 0.60; *δ_I_* = −0.28 and, therefore, the bias that arose when this model was applied was very small. On the other hand, comparisons using the estimates obtained using Unit D's data produced large values for the ratio of SMRs (1.11 to 1.68) because the model estimates were very different to the published estimates: *α_D_* = 11.59; *β_D_* = −0.56; *γ_D_* = 1.64; *δ_D_* = −0.11.

**Figure 2:**
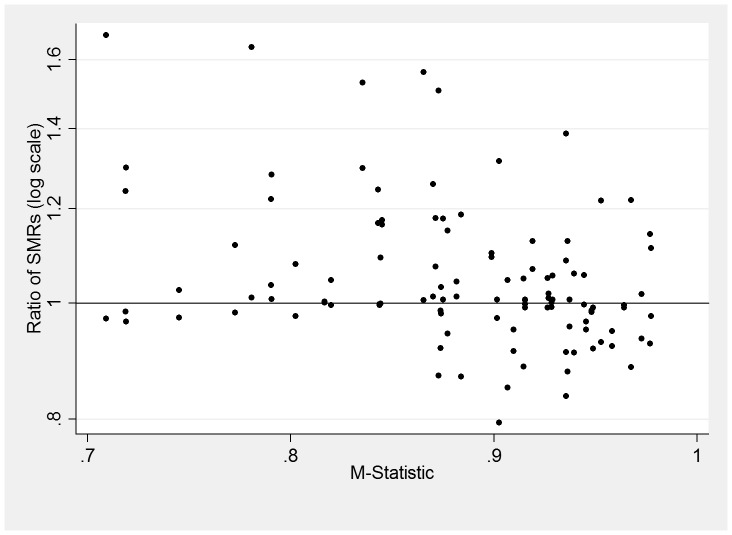
Plot of the M-statistic for each pair of units and both of the corresponding ratios of the SMRs for the pair of units.

M-statistics were then calculated for each pair of units and were plotted against their corresponding ratio of SMRs (Figure 2). There was no clear pattern in the relationship between the value of the ratio of the SMRs and the value of the M-statistic: 63 of the values for the ratio were greater than 1 and 47 less were than 1. There was an absence of low values for the ratio when the value of the M-statistic was low predominately due to Unit D which had a quite different case-mix to the other neonatal units (producing the low value for the M-statistic) but generally lower risk-specific probabilities of death.

## Discussion

In this paper the potential bias associated with using SMRs to directly compare case-mix adjusted mortality of very preterm babies between two neonatal units has been described and quantified. This problem is a form of Simpson's Paradox [31]; even when two neonatal units are known to have the same risk-specific probabilities for mortality, it is possible to observe differences in their SMRs. The size of this bias is dependent on the difference in the case-mix structure of the units and the variation from the risk-specific probabilities of mortality observed in the reference population.

The observed bias tended to be small when comparing Network Neonatal Units as their populations were more similar than when comparing across all units, since they admit similar proportions of high risk babies and have generally similar patterns of transfers. In principle, pairs of units with a high value for the M-statistic would be expected to have values for the ratio of SMRs closer to the value 1, as units with very high M-statistics had similar proportions of babies at the different levels of risk. However, in this research the M-statistic alone did not appear to be a reliable diagnostic tool for predicting the bias. Even for values of the M-statistic above 0.9, five pairs of SMRs showed differences of over 20% (Figure 2). This was due to the variation from the risk-specific probabilities of death observed in the reference population and this would also need to be taken into account in any tool to predict which comparisons would likely be misleading.

Alternative summary statistics have been suggested which, unlike the SMR, do allow direct comparison between two healthcare providers. The Comparative Mortality Figure (CMF) is obtained by direct standardisation; the ratio of the number of events observed in the reference population to the number expected calculated using the risk-specific probability of the event for the healthcare provider [32]. When using the CMF, all providers are standardised to the same reference population and, therefore, meaningful direct comparisons can be made between healthcare providers. However, since this method requires the reliable estimation of risk-specific event probabilities for each provider it is unlikely to be appropriate except when there are a large number of events. Other statistics have been suggested such as the Harmonically Weighted Ratio (HWR) [33] and the Geometrically Averaged Ratio (GAR) [34]. However, unlike the SMR or the CMF these statistics do not have an intuitive interpretation and are, therefore, less suitable for routine use.

In practice this research shows that when comparing neonatal survival of preterm babies the direct comparison of SMRs from units providing different levels of care could lead to misleading conclusions. We have only investigated the potential bias under the assumption of no difference in the risk-specific mortality probabilities between units. In reality, these probabilities will differ between units but this bias could alter, and perhaps reverse, these real differences in the SMRs. It is important when directly comparing SMRs for neonatal mortality that comparisons between units of different types should be undertaken with particular caution. The same caution should be applied when directly comparing between Local Neonatal Units as these also appear to differ in case-mix sufficiently to introduce bias to direct comparisons. The size of the bias that would lead to any comparison being misleading obviously depends on the purpose for which the comparison is being made. However, it is likely that observed differences in SMRs that are 20% greater than the ‘true’ difference would need to be interpreted with caution.

### Limitations

The main limitation of this work is that the estimates for the ‘reference’ unit specific model parameters for Local Neonatal Units were derived from small samples with few deaths. This means that it is possible that the estimated risk-specific probabilities of death from these models could be implausible for some groups of babies, especially where the model was extrapolated beyond observed data. Since the size of the potential bias present when comparing two SMRs is dependent on the difference between the risk-specific probabilities of death in the reference group and those observed in the unit of interest, any extreme estimates derived for the Local Neonatal Units may have resulted in overestimates of the likely values of the bias found in practice. This may be the case with the model developed from Unit D, although the values of the parameter estimates appeared plausible on inspection.

This work is based on a relatively small number of neonatal units and the results obtained may not necessarily be generalizable to other neonatal units. However, there is no evidence to suggest that the organisation and practice of these units is different from that of other UK neonatal units.

### Conclusion

If the populations of healthcare providers differ considerably then it is likely that bias will be an issue when directly comparing SMRs. In neonatal care, the comparison of Network Neonatal Units is likely to be appropriate but caution is required when comparing between Local Neonatal Units as these differ considerably in case-mix from each other. More generally it is important that there is a proper understanding of whether a single risk model should be used for comparing any group of service providers.

Future work is required to develop a better tool than the M-statistic to quantify the likely bias.
